# Supplementary material for: Mothers’ hygiene experiences in confinement centres: A cohort study
Source: PLoS One. 2022 May 23;17(5):e0268676. doi: 10.1371/journal.pone.0268676 (PMC9126405; doi:10.1371/journal.pone.0268676)
Supplement: S1 File — (PDF) [file pone.0268676.s002.pdf]

General Questions

1. Where did you spend your confinement period? (If both, mother to specify duration in each and reasons for the change?
2. Who is the primary caretaker of your baby during the confinement period?
3. Did you baby sleep with you during your confinement period? (If not, why?)
4. In the last week of your confinement period, approximately how much time did you spend with your baby in a day (both day and night)?

Hygiene questions (General)

1. How was your baby's general health during the confinement period?

Hygiene questions for mothers who employed a traditional postpartum carer at home:

1. Did the TPC wash her hands before touching the baby or after changing diapers?
2. If she did not wash her hands, what did you do about it?
3. Did the TPC stay with you throughout your confinement period? If not, approximately how many days did she stay with you and why did she leave early?

Hygiene questions for mothers staying in confinement centres (CC):

1. How many mothers were there in your room including yourself?
2. Were there a maximum number of mothers your CC would accept?
3. Do you know how many rooms were available for mothers in your CC?
4. How many nurseries where there for babies in your CC?
5. How was the overall cleanliness of your CC? (Scale of 0 to 3: Very clean [3], clean [2], somewhat dirty [1], very dirty [0])
6. Were there any areas in your CC that was not up to your expectations? (Please elaborate why it was not up to your expectations)
7. Was there a hand basin in your room?
8. Was there hand soap provided at all basins that you use? (Please elaborate where necessary)
9. Was there anything provided for you to wipe your hands? (Please describe)
10. Were alcohol hand sanitizers available?
11. How many babies were there in each nursery?
12. Did the CC staff wash hands between handling babies? (If no, what did you do about it?)
13. Did the CC have quarantine rooms for the babies if they fall sick?
14. Did the CC have quarantine rooms for the mothers if they fall sick?
15. Is there anything else positive about your CC that you would like to share?
16. Is there anything else negative about your CC that you would like to share?

Breastfeeding questions (General)

1. During your confinement, how did you mainly feed your baby? (If not breastfeeding, ask why)
2. (If mother is breastfeeding): How are you mainly breastfeeding? (ie direct latch or expressed milk feeding only or both?)
3. (If only expressed milk feeding only): Why don't you breastfeed directly from breast?
4. (If applicable): Who was the main person to feed your baby with expressed breast milk?
5. (If applicable): What was used to feed the expressed milk to your baby? (eg cup or bottle)
6. When do you feed your baby? (Read out options: On demand eg whenever baby seems hungry, strict schedule ie not feed if it's not the scheduled time, I don't know because feeding is done by other people, others (elaborate).)

7. (If baby is not fed on demand). Why?
8. Did you encounter any problems during breastfeeding in your confinement period?
9. Where did you get help from when you faced those problems?
10. How satisfied were you with the help obtained? (On a scale of 0 to 4, with 1 being not helpful at all, and 4 being very helpful)
11. (If mother's response is category 0 or 1): Can you tell us why?
12. Did anyone make it difficult for you to breastfeed?

Breastfeeding questions for mothers staying in CCs

1. Was your CC supportive of breastfeeding? If yes, what support was given?
2. How supportive do you think the CC is? (On a scale of 0 to 4, with 1 being not supportive at all, and 4 being very supportive towards breastfeeding
3. (If category 0 or 1), can you elaborate why you said so?
4. Did your CC allow baby to sleep with you?
5. Did the CC allow you free access to the nursery to see your baby any time of the day?
6. Did your CC allow you to breastfeed any time of the day including the middle of the night?
7. Was it the practice of your CC to give your baby a bottle (of formula) at night?
8. (If you expressed breast milk) How was your milk kept?
